# Embolization of feeding arteries and symptom alleviation of mixed dural-pial arteriovenous malformations

**DOI:** 10.1186/s41016-018-0111-1

**Published:** 2018-02-27

**Authors:** Hengwei Jin, Hong Qiu, Chang Chen, Huijian Ge, Youxiang Li, Hongwei He

**Affiliations:** 1 Department of Interventional Neuroradiology, Beijing Neurosurgical Institute and Beijing Tiantan Hospital, Capital Medical University, No.6, Tiantan Xili, Dongcheng, Beijing 100050 China; 2 Neurosurgery Department, Xintai Hospital of Traditional Chinese Medicine, Tai’an, Shandong 271200 China; 3 Beijing Engineering Research Center for Interventional Neuroradiology, No.6, Tiantan Xili, Dongcheng, Beijing 100050 China

**Keywords:** Mixed dural-pial arteriovenous malformations, Endovascular treatment, Clinical outcome

## Abstract

**Background:**

To examine whether embolization of dural or pial blood supply branch is more efficient for symptom alleviation for unruptured mixed dural-pial arteriovenous malformations (DPAVMs).

**Methods:**

We retrospectively reviewed 30 DPAVM patients from a database of 425 consecutive cerebral arteriovenous malformation (CAVM) patients who underwent endovascular embolization between January 2010 and December 2015 at our institution. Demographics, angioarchitectural characteristics, endovascular embolization details and patients clinical outcomes were recorded. The modified Rankin Scale (mRS), Engel‘s classification and Visual Analogue pain scale (VAS) were used to assess clinical outcomes.

**Results:**

The single center cohort data shows that the incidence of DPAVM is 7.1%. Among the 30 DPAVM patients, 9 (30.0%) are ruptured and 21 (70.0%) are unruptured. Four (19.0%) of the 21 unruptured DPAVM patients are failed to follow-up, leaving 17 to analysis the clinical outcomes. Clinical presentations of the 17 unruptured DPAVM patients are epilepsy (*n* = 10), headache (*n* = 5) and focal neurological dysfunction (*n* = 2). Six patients have DPAVMs occluded via pial blood supply branches, 4 via dural branches and 7 via both pial and dural branches. Unruptured DPAVM patients with nidus occluded via dural blood supply branches, or both pial and dural branches have higher symptom alleviation rate than patients with nidus occluded via pial branches (100%/85.7% vs 66.7%).

**Conclusions:**

For DPAVM patients presented with epilepsy, headache and FND, embolization via dural blood supply branches may be more efficient for symptom alleviation. Large cohort study is needed to confirm the generalizability.

## Background

In 1969, Newton classified cerebral arteriovenous malformation (CAVM) into pure pial, mixed dural-pial and pure dural types, and analyzed 20 mixed dural-pial arteriovenous malformation (DPAVM) patients. DPAVM frequently received a portion of blood supply from dural meningeal arteries. The dural contribution may arise from meningeal branch of the internal carotid, the external carotid and the vertebral arteries [1]. Pial CAVMs were generally thought to be congenital, whereas dural CAVMs were acquired. Acquired dural CAVMs were well known to develop secondary to sinus thrombosis or trauma [1–4].

The treatment of CAVMs is based on an objective comparison of natural risks with operative treatment risks. DPAVMs are usually large in size and complicated [5]. The treatment of DPAVMs usually requires a combination of three therapeutic modalities, including endovascular therapy, microsurgery and stereotactic radiosurgery. The cure rate is extremely low, and morbidity and mortality is high [6, 7]. Moreover, ARUBA showed that medical management is superior to medical management with interventional therapy for the prevention of death or stroke in patients with unruptured CAVMs [8]. On conditions that complete obliteration or resection is less feasible and that symptoms confuse patients persistently, how to alleviate or eliminate symptoms such as epilepsy, headache and focal neurological dysfunction (FND) seems important for unruptured DPAVM patients. Both dural and pial branches have blood supply to the nidus in DPAVM, whether the occlusion of dural or pial artery branch is more efficient for symptom alleviation is not well known.

In this retrospective study, we compared the symptom alleviation rate of DPAVM patients presented with epilepsy, headache or FND, to examine whether dural or pial blood supply artery branch occlusion is more efficient for symptom alleviation.

## Methods

### Patients

A series of 425 consecutive CAVM patients underwent endovascular treatment between January 2010 and December 2015 at Beijing Tiantan hospital were retrospectively reviewed. Two neurointerventional radiologists reviewed angiography of the cohort independently to confirm the lesion and presence of a dural blood supply. A deep CAVM was defined as one involving ventricular nuclei, thalami, ventricles or diencephalon. A cortical CAVM was defined as one on the surface of the cerebrum. An infratentorial CAVM was defined as one on the brainstem or cerebellum. Patients received structured telephone follow-up. Demographics, angioarchitectural characteristics, procedural details, and clinical outcomes at follow-up were recorded.

### Endovascular treatment

Treatment indications for unruptured patients include high hemorrhage risks (aneurysm, high flow fistula or venous porch) and/or uncontrolled or progressive symptoms. Endovascular embolization procedures were performed intra-arterially via a transfemoral route with the patients under general endotracheal anesthesia. Systemic heparinization was initiated before microcatheter navigation to achieve an activated clotting time 2–3 times of the normal. Superselective catheterization of the arterial pedicles supplying the nidus was performed by using flow-directed microcatheters (Marathon, ev3). Embolization was performed by using either n-butyl cyanoacrylate (NBCA) or ONYX (Onyx 18, Ev3) when microcatheter tip was as close as possible to the nidus. If aneurysms were noted during selective injection, the initial treatment was concentrated on the aneurysms,followed by the nidus. The technique remained the same throughout the study period.

### Functional assessment

We used the modified Rankin Scale (mRS) to assess the functional outcome of patients at the last available follow-up. We used Engel‘s classification to assess the outcome of patients presented with epilepsy [9], and Visual Analogue pain scale (VAS) to assess the outcome of patients presented with headache [10]. For patients who received microsurgery or radiological therapy after embolization, the clinical outcome is the immediate functional state before microsurgery or radiological therapy. We defined symptom relief as a grade reduction after interventional therapy on condition that the patients received the same medical therapy as pre-operation or less medical therapy than pre-operation. Complications were classified into hemorrhagic, transient ischemic and permanent ischemic categories. Hemorrhagic complication was defined as intracerebral hemorrhage (ICH) happened within 1 week after embolization. Ischemic complications were defined as additional dysfunction or worsened previous dysfunction.

## Results

A total of 30 (7.1%) DPAVM patients were identified. Among the 30 DPAVM patients, 9 (30.0%) are ruptured and 21 (70.0%) are unruptured at initial presentation. Demographic and angioarchitectural characteristics of all patients are listed in Table 1. Among 21 unruptured patients, 4 were failed to follow up, leaving 17 to analyze the clinical outcome. Follow-up period ranges from 12 to 48 months (mean ± SD: 25.3 ± 10.4).

**Table 1:** Demographic and angioarchitectural characteristics of 30 DPAVM patients

Characteristics	Number of patients (%)
Age at presentation, years, Mean(±SD)	28.3(±9.7)
Gender, Male	13(43.3)
Symptom	
Hemorrhage	9(30.0)
Epilepsy	11(36.7)
Headache	6(20.0)
FND	4(13.3)
Location	
Cortical location	28(93.3)
Deep	1(3.3)
Infratentorial	1(3.3)
Eloquent area	11(36.7)
Origin of dural branch involved in blood supply	
ECA	29(96.7%)
VA	1(3.3%)
Size	
<3 cm	2(6.7)
3-6 cm	12(40.0)
>6 cm	16(53.3)
Deep Venous drainage	6(20.0)
SM Grade	
I-II	10(33.3)
III-IV	19(63.3)
V	1(3.3)
Coexisting aneurysms	2(6.7)
Coexisting DAVF	10(33.3)

*DPAVM* mixed dural-pial arteriovenous malformation, *SD* standard deviation, *FND* focal neurological dysfunction, *ECA* external carotid artery, *VA* vertebral artery, *SM* Spetzler-Matin Grading system, *DAVF* dural arteriovenous fistula

**Fig. 1:**
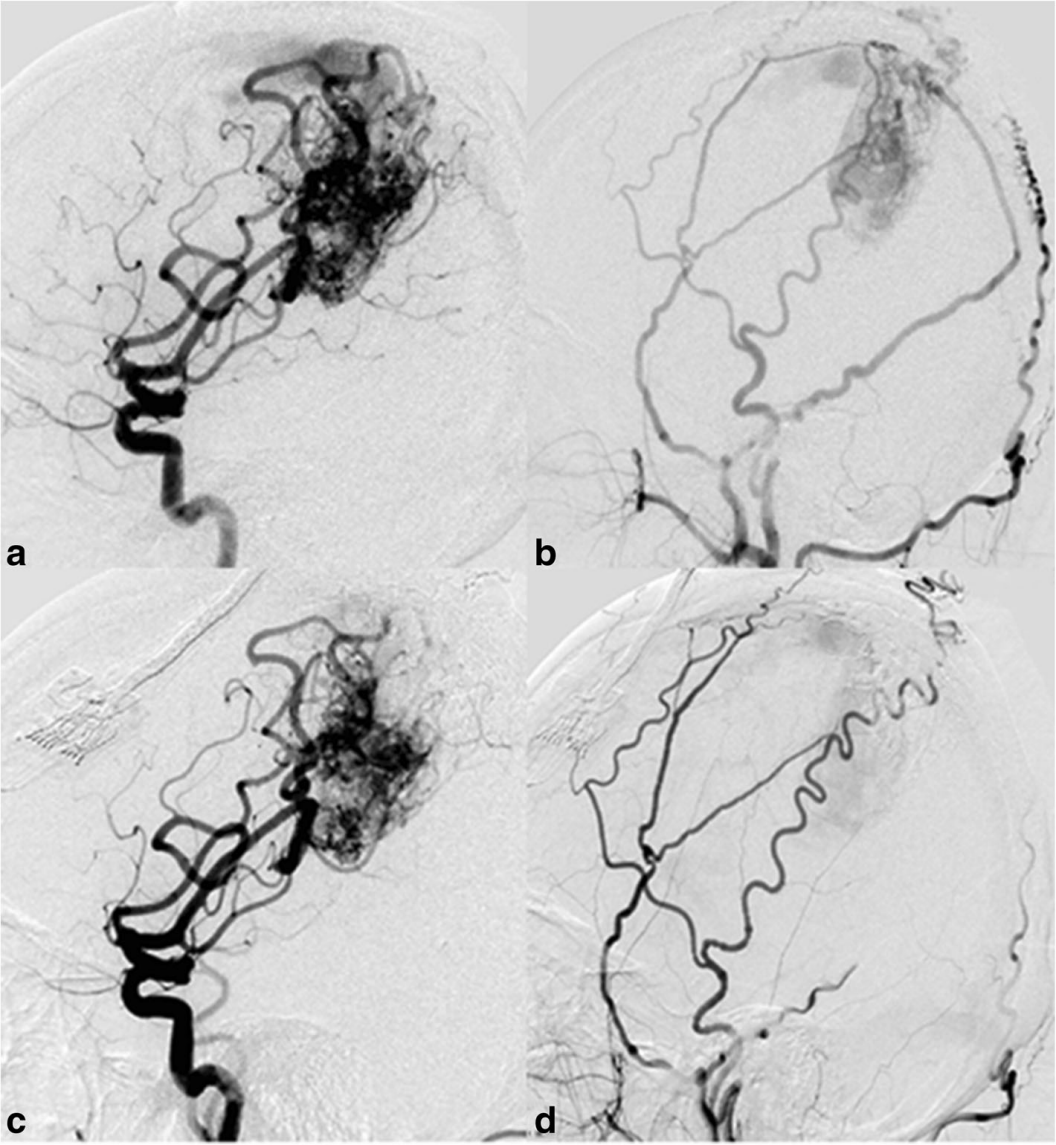
A 33 years old woman with chief complaint of epilepsy for 2 weeks. **a**: Lateral view of right internal carotid artery angiography before embolization reveals right parietal arteriovenous malformation supplied by branches of right middle cerebral artery. **b**: Lateral view of right external carotid artery angiography before embolization. Both anterior and posterior branches of right middle meningeal artery are involved in blood supply. **c**: Lateral view of right internal carotid artery angiography after embolization. The nidus is partly embolized. Branches of right middle cerebral artery still supply part of the nidus. **d**: Lateral view of right external carotid artery angiography after embolization. Blood supply of right middle meningeal artery is completely eliminated

A total of 25 sessions (1.5 per person) of embolization were performed on the 17 patients. In all, 32 (1.3 per session, 1.9 per person) pedicles were occluded. In the 17 patients, 4 were occluded via dural blood supply branches, 6 via pial blood supply branches and 7 via both dural and pial branches. (Fig. 1) Compared with patients who were occluded via pial blood supply branches, patients via dural blood supply branches had higher symptom alleviation rate (100% vs 66.7%). The symptom alleviation rate of patients via both dural and pial branches (85.7%) was also higher than that of patients via pial branches (Table 2).

**Table 2:** Occlusion artery and outcomes of 17 unruptured DPAVM patients

Symptom	Occlusion artery			
	Pial, /n	Dural, /n	Both, /n	Total, /n
Epilepsy relieved or disappeared, n/	3/4	2/2	3/4	8/10
Headache relieved or disappeared, n/	1/2	2/2	1/1	4/5
FND relieved or disappeared, n/	0/0	0/0	2/2	2/2
Total, n/	4/6	4/4	6/7	14/17

*DPAVM* mixed dural-pial arteriovenous malformation, *FND* focal neurological dysfunction

**Fig. 2:**
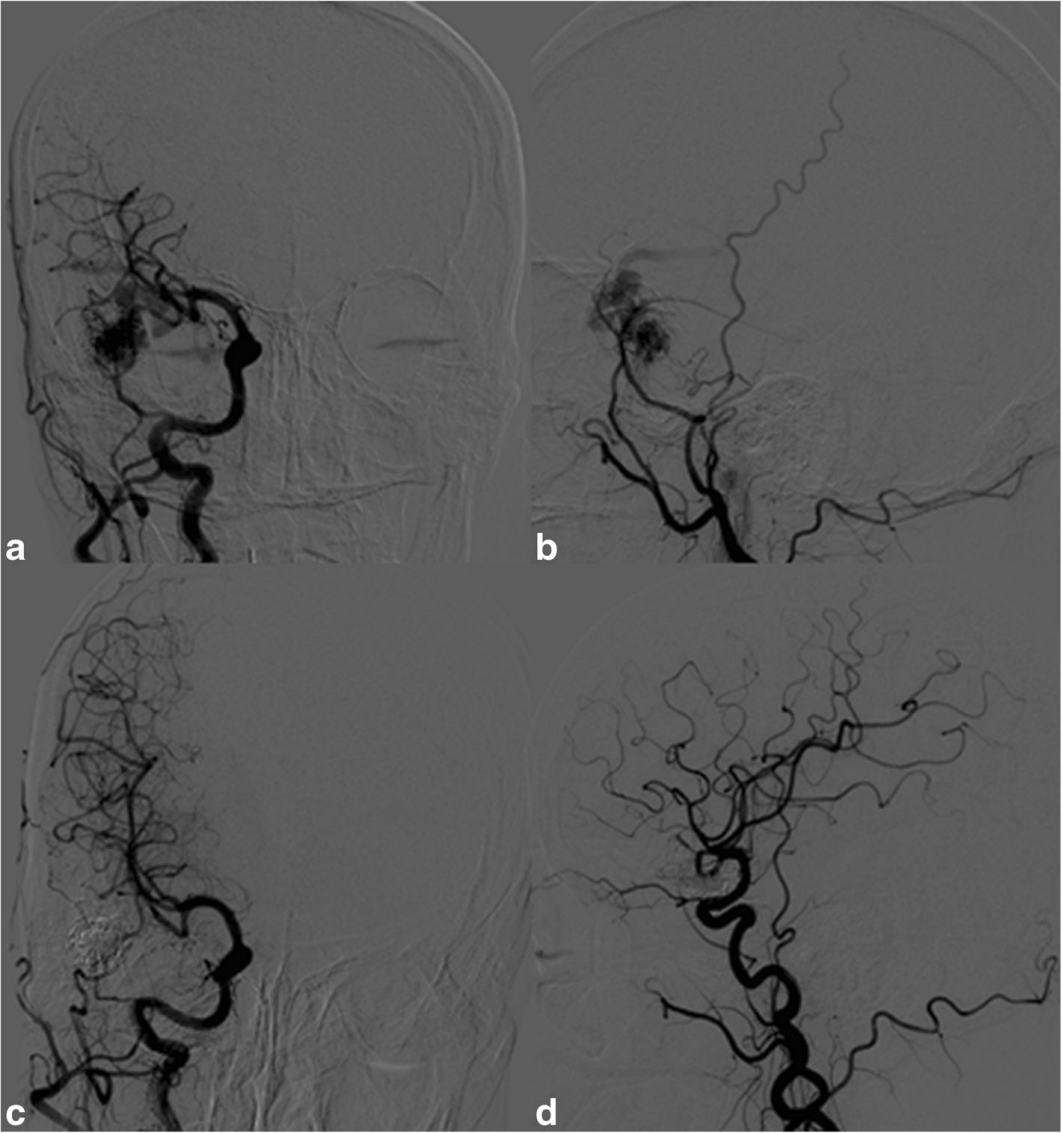
A 49 year old woman with main complaint of epilepsy for half a year. **a**: Anteroposterior view of right carotid artery angiography before embolization reveals right temporal arteriovenous malformation supplied by temporal branch of right middle cerebral artery. **b**: Lateral view of right external carotid artery angiography before embolization. Anterior branch of left middle meningeal artery is involved in blood supply. **c** and **d**: Anteroposterior and lateral view of right carotid artery angiography after embolization shows that the nidus is completely occluded

Two (11.7%) of the 17 DPAVMs (10.0%) are completely occluded. (Fig. 2) One (5.9%) patients experienced transient ischemic complication and completely recovered (mRS = 0) at discharge. The outcome of this patient is accessed at 20 months after embolization. No patients experienced hemorrhagic or permanent ischemic complications resulted by procedure. One (5.9%) patients experienced ICH one and a half years after embolization. The outcome of this patient is accessed before ICH. Two (11.7%) patients underwent microsurgery and 6 (35%) patients underwent radiological therapy after embolization.

## Discussion

In this retrospective study, we discovered that symptom alleviation rate is higher in patients who were embolized via dural branches or both dural and pial branches than patients who were embolized via pial branches. The potential reason may be that the stimulations, which are caused by pulsatile dural feeding artery, exerted on dura and cortex are relieved. The result of our cohort could remind us that dural blood supply artery approach may have advantage over pial blood supply artery approach in symptom alleviation of unruptured DPAVM patients presented with epilepsy, headache or FND.

The incidence of CAVM is around 1.1–1.3/100,000 [11, 12]. Participation of dural meningeal branches in the supply of CAVM is relatively uncommon. In 1958, Krayenbuhl and Yasargil [13] noted dural contribution in 1 of 90 patients with CAVM. Newton analyzed 129 CAVMs and 20 were DPAVMs [1]. The incidence rate of DPAVMs in the current study is 7.1%. The pathogenesis of mixed DPAVM is still controversial. The presence of abnormal communications between the pial and dural arteries in the embryonic stage may lead to the development of mixed DPAVM [6]. Venous hypertension secondary to sinus thrombosis is believed to be the primary mechanism responsible for the formation of DPAVM. Lawton speculated that increased cortical venous pressure might decrease cerebral perfusion and increase ischemia, and that dural CAVM formation might be the result of aberrant angiogenesis [14]. Large CAVMs are more likely to develop into DPAVMs. This may because large nidus contains more concealed abnormal communications between the pial and dural arteries, which would undoubtedly provide more chances for the formation of dural-pial CAVM when venous hypertension happens [5]. More than 50.0% of the nidus in this study are >6 cm and 93.3% are >3 cm, which is consistent with previous studies.

Clinical presentations of CAVMs are ICH, epilepsy, headache and FND. The most common presentation is ICH and seizure is the second most common symptom [15]. Tong et al. reported an analysis of 3299 CAVM patients in china, among which 57.9% presented with hemorrhage, 20.9% with epilepsy and 14.9% with chronic headache [16]. In this study, the percentage of hemorrhage presentation (30.0%) is lower and epilepsy presentation (36.7%) is higher. Deep and infratentorial location is indication of CAVM hemorrhage [17, 18]. The percentage of deep and infratentorial location DPCAM in this cohort is small, so it is logically feasible that the percentage of hemorrhage presentation is small. It seems that DPAVM patients are more inclined to present with epilepsy. This may because DPAVM group has higher percentage of cortical located nidus. Nidus that are more adjacent to cortical may stimulate pial mater and cerebral cortex, which will cause abnormal discharge. Moreover, pulsatile veins and obstruction of venous efflux caused by venous hypertension may increase the incidence of seizures [19].

This is a retrospective study. The generalizability of our results is limited by the small sample size. There are only a few cases in each category based on various occlusion arteries. We could only describe with frequencies and our conclusion still needs a large prospective study to confirm.

## Conclusions

For DPAVM patients presented with epilepsy, headache and FND, embolization via dural blood supply branches may be more efficient for symptom alleviation. Large cohort study is needed to confirm the generalizability.

## Abbreviations

CAVM: Cerebral arteriovenous malformation
DAVF: Dural arteriovenous fistulas
DPAVM: Dural-pial arteriovenous malformation
ECA: External carotid artery
FND: Focal neurological dysfunction
ICA: Internal carotid artery
ICH: Intracerebral hemorrhage
SM: Spetzler Martin grade
